# Spatial distribution and predictors of intimate partner violence among women in Nigeria

**DOI:** 10.1186/s12905-022-01823-w

**Published:** 2022-06-20

**Authors:** Obasanjo Afolabi Bolarinwa, Bright Opoku Ahinkorah, James Boadu Frimpong, Abdul-Aziz Seidu, Zemenu Tadesse Tessema

**Affiliations:** 1 Department of Global Public Health, Canterbury Christ Church University, Canterbury, CT1 1QU United Kingdom; 2 Department of Public Health Medicine, University of KwaZulu-Natal, Durban, 4049 South Africa; 3 School of Public Health, University of Technology Sydney, Sydney, NSW 2007 Australia; 4 Department of Health, Physical Education and Recreation, University of Cape Coast, PMB TF0494, Cape Coast, Ghana; 5 Centre for Gender and Advocacy, Takoradi Technical University, Takoradi, Ghana; 6 College of Public Health, Medical and Veterinary Sciences, James Cook University, Townsville, QLD 4811 Australia; 7 Department of Epidemiology and Biostatistics, Institute of Public Health, College of Medicine and Health Sciences, University of Gondar, Gondar, Ethiopia

**Keywords:** Spatial distribution, Predictors, Intimate partner violence, Nigeria

## Abstract

**Background:**

Globally, intimate partner violence is one of the major health problems women face every day. Its consequences are enormous. However, our search of the available literature revealed that no study had examined the spatial distribution of intimate partner violence and the predictors of intimate partner violence among women in Nigeria using current nationally representative data. This study, therefore, sought to examine the spatial distribution of intimate partner violence and its predictors among women in Nigeria.

**Method:**

We sourced data from the 2018 Nigeria Demographic and Health Survey for this study. A sample size of 8,968 women was considered for this study. We employed ﻿both multilevel and spatial analyses to ascertain the factors associated with intimate partner violence and its spatial clustering.

**Results:**

The hot spot areas for intimate partner violence in Nigeria were Gombe, Bauchi, Adamawa, Plateau, Kogi, Edo, Ebonyi, and Rivers. The likelihood of experiencing intimate partner violence among women in Nigeria was high among women with primary education, those that were previously married, women currently working, women who were Yoruba, women with parity of four and above and women who were exposed to mass media while low odds of intimate partner violence was reported among women who were Muslims. Women who resided in the North East region and those who lived in communities with medium socioeconomic status were more likely to experience intimate partner violence, while women who were within the richest wealth index and those residing in the South West region were less likely to experience intimate partner violence.

**Conclusion:**

The study found regional variations in the prevalence of intimate partner violence among women in Nigeria. Therefore, policymakers should focus their attention on the hotspots for intimate partner violence in the country. There is also the need to consider the factors identified in this study to reduce intimate partner violence among women in Nigeria. Empowering women would yield a significant improvement in the fight against gender-based violence.

## Background

Intimate partner violence (IPV) is one of the major health problems that women face [1, 2]. IPV is a situation where an individual subjects a spouse or a partner in a romantic relationship to physical, sexual, emotional, or psychological torture [3, 4]. IPV is gender-based violence with no boundary in terms of nationality, religion, culture, or class [5, 6]. The World Health Organisation (WHO) estimated in 2013 that every one in three women in the world has ever been beaten, forced into sexual intercourse or abused in their lifetime [1], this estimate still persist in the recent report published in 2021 and got exacerbated with the advent of 2019 Coronavirus disease (COVID-19) [7]. A common form of violence perpetrated against women is abuse from their intimate partner [5].

IPV could have devastating effects on the physical and mental health of victims. For instance, IPV affects the reproductive health of women, which may lead to an increase in their likelihood of having miscarriages, pregnancy termination, stillbirths, induced abortions, unplanned pregnancies, and pregnancy-related complications [6, 8–11]. Aside from the instant or short-term traumatic consequences, IPV can result in chronic health problems such as physical disability, drug and alcohol abuse and addiction, depression, suicidal ideation, and even death [4, 12, 13].

Despite the devastating effects of IPV on the health of women, the family cultural norms in Nigeria which has been reported to be oligarchy and patriarchy have seemingly favored perpetrators of gender-based violence, making victims shy away from reporting such issues to law enforcement agencies and health care providers [5, 12, 14, 15]. Previous studies conducted in Nigeria have shown that factors such as employment status, educational attainment, spouse’s educational attainment, religion, marital status, and wealth status were predictors of IPV among women [8, 16, 17]. In the same vein, most of the available studies indicated that the prevalence of IPV among women in Nigeria is high [3, 8, 18]. For example, Benebo, Schumann, and Vaezghasemi [19] reported that one out of four women in Nigeria had ever experienced IPV [19]. Therefore, it is important to focus more research attention on the factors that predict the perpetration of IPV against women, especially in recent times.

Up until now, our search of the available literature revealed that none of the studies had examined the spatial distribution of IPV and the predictors of IPV among women in Nigeria using current nationally representative data from the Nigeria Demographic and Health Survey (NDHS) [20, 21]. As a result, designing and implementing policies specific to Nigeria's various regions or clusters has become a daunting task for policymakers. This creates a significant gap in extant literature that needs urgent research attention. Therefore, this study examined the spatial distribution and predictors of IPV among women in Nigeria using the recent NDHS data conducted in 2018. Findings from the study could help direct policies that would help reduce gender-based violence against women, which is in line with the United Nations’ (UN) Sustainable Development Goal (SDG) 5 of achieving gender equality and empowering women and girls by the year 2030 [22].

## Methods and materials

### Data source

This is a cross-sectional analysis of population-based data from the 2018 NDHS. The NDHS is a nationally representative survey used to gather sociodemographic and other health-related indicators such as IPV [23]. A two-stage sampling procedure was used to gather data from 36 administrative units and the Federal Capital Territory (FCT). The survey's primary sampling unit was made up of samples drawn at random from clusters. A total of 41,821 women aged 15–49 participated in the 2018 study. From this number, 8968 women who had complete information and participated in the domestic violence module were considered in this study. The sampling, pretesting and the general methodology of the 2018 NDHS have been published elsewhere [24, 25]. In writing this manuscript, we adopted the guidelines for improving the reporting of observational studies in Epidemiology [26]. The dataset utilised in this study is available in the public domain and can be downloaded from https://dhsprogram.com/data/available-datasets.cfm.

### Dependent variable

IPV was the outcome variable. It was obtained from the following variables: sexual violence, emotional violence, and physical violence. These three variables were derived from a series of questions in the domestic violence module that were related to a variety of violent acts that a woman had experienced. Previous studies include details on the questions for each aspect of three forms of IPV [27, 28]. There were Yes, or No response questions asked for each element of IPV. Therefore, a woman who had undergone at least one of the acts was regarded as ever experienced physical, emotional, or sexual abuse. From the questions asked on the experience of physical, emotional, and sexual abuse, IPV was created with respondents experiencing at least one of these violent acts regarded as ever had IPV and otherwise [9, 29].

### Independent variables

Based on theoretical and practical significance and the availability of the variables in the dataset, we considered both individual and contextual factors in our study [23]. These were also influenced by their association with IPV in several previous studies in Nigeria and sub-Saharan (SSA) in general [9, 17].

- The individual-level factors were age (15–24, 25–34, 35+), educational level (No education, primary, secondary/higher), husband/partner’s educational level (No education, primary, secondary/higher), marital status (currently married, cohabiting, previously married), working status (not working, working), ethnicity (Hausa, Yoruba, Igbo, Others), religion (Christianity, Islam, Traditionalist & Others), parity (0, 1–3, 4+), and exposure to mass media (yes, no) [9, 17].
- The contextual factors were place of residence (urban and rural), wealth index (poorest, poorer, middle, richer, richest), region (North Central, North East, North West, South East, South South, South West), sex of household head (male, female), community literacy level (low, medium, high), community socioeconomic status (low, medium, high) [9, 17].

#### Analyses

We employed both spatial and multilevel analyses in analyzing the data.

#### Spatial analysis

Different statistical software like Excel, SaTScan, ArcGIS, and Stata 16 were utilized for spatial distribution of IPV in Nigeria. A total of 1400 clusters or Enumerations Areas (EAs) were considered for this study. Among these clusters, seven were dropped because they had no measured longitude and latitude data. The data were weighted with v005 (weighing variable) and geographic coordinate data were merged in Stata 16 and then exported to excel, which was finally imported to ArcGIS 10.7 for spatial analysis.

### Spatial autocorrelation

To check whether there is clustering effect in IPV in Nigeria, spatial autocorrelation analysis was done. This analysis result gives Global Moran’s I value, Z-score and p-value for deciding whether the data is dispersed or random or clustered. Moran’s I value close to positive 1 indicates there is clustering effect, close to negative one indicates dispersed and close to zero indicates random. If p-value is significant and Moran’s I value is close to mean, that means IPV had clustering effect [30].

### Hot spot

The hot spot analysis tool gives a Getis_Ord or Gi* statistics for cluster in the dataset. Statistical values like Z-score and p-value is computed to determine the statistical significance of the clusters. Results of the analysis with high GI* value means hot spot areas and low GI* value means cold spot areas [31].

### Prediction of IPV

Spatial prediction is one of the techniques of furcating unsampled areas based on sampled areas. In Nigeria, a total of 1400 enumeration areas were selected to take a sample for this areas that believed to be representative of the country. A total of seven clusters had no enumeration longitude and latitude were dropped. Based on 1393 sampled areas, it is possible to predict the remaining parts of Nigeria. Ordinary Kriging prediction methods were used for this study to predict IPV in unobserved areas of Nigeria.

### SaTScan analysis result

Bernoulli purely spatial model was applied to identify IPV clusters using 1393 enumeration areas. SatTscan Software was used for the analysis. First, the dataset was managed as appropriate for the SaTScan software. Women who faced IPV were taken as cases and women who did not face IPV were taken as controls. The Cluster number, longitude and latitude data were obtained from GPS dataset. The cluster size less than 50% of the population was taken as upper bound. A Monte Carlo replication was used for this study. Based on the above criteria, primary clusters were identified.

### Statistical analysis

#### Multilevel analysis

A two-level multilevel binary logistic regression models were fitted to evaluate the individual and contextual (household and community level) factors linked to IPV experience among women in Nigeria. In the modelling, women were nested within households; then households were nested within clusters. To account for the unexplained variability at the community level, clusters were proposed as a random effect. A total of four models were fitted. Firstly, we fitted an empty model, model 0, which contained no predictors (random intercept). Following that, model I only included individual-level variables, model II only included contextual-level variables, and model III included both individual-level and contextual-level variables. The odds ratio and related 95% confidence intervals were provided for all models. These models were fitted using a Stata command “melogit” for the identification of predictors with the outcome variable (IPV). The log-likelihood ratio (LLR), Akaike Information Criteria (AIC) was used to compare models. The best fit model has the highest log-likelihood and the lowest AIC [32]. The multicollinearity test, which used the variance inflation factor (VIF), revealed no evidence of collinearity among the independent variables (Mean VIF = 1.83, Maximum VIF = 1.17, and Minimum VIF = 3.09). The domestic violence module sample weight (d005/1,000,000) was used in all analyses to account for over-and under-sampling, while the svy command was used to account for the complex survey design and generalizability of the results. All the analyses were carried out using Stata version 16.0 (﻿Stata Corporation, College Station, TX, USA).

### Ethical approval

Since the authors of this manuscript did not collect the data, we sought permission from the MEASURE DHS website and access to the data was provided after our intent for the request was assessed and approved on the 6th of April 2021. The DHS surveys are ethically accepted by the ORC Macro Inc. Ethics Committee and the Ethics Boards of partner organizations in different countries, such as the Ministries of Health. All methods were performed in accordance with the relevant guidelines and regulations. The women who were interviewed gave informed consent during each of the surveys.

## Results

### Socio-demographic characteristics of respondents

A total weighted sample of 8968 women was included in the study. At the individual level, 3807 (42.44%) of the respondents were aged 25–34, 4154 (46.31%) of women had secondary education and above, 4697 (52.37%) of husbands/partners had secondary school and above, 8128 (90.63%) were currently married, 6472 (72.16%) were currently working, 3046 (33.97%) of women ethnicity was Hausa, 4667 (52.03%) of the respondents practiced Islam, and 6183 (68.94%) had mass media exposure. At household/community level, 4905 (54.69%) of women resided in the rural area, 1952 (21.76%) were from the richest household, 2434 (27.14%) were residing in North West, 3124 (34.83%) were from a community with high literacy level, and 5131 (57.21%) were from a community with low socioeconomic status (Table 1).

**Table 1:** Individual and household-level characteristics of respondents

Variable	Weighted Frequency	Weighted percentage
Age of respondent (years)		
15–24	1828	20.39
25–34	3807	42.44
35 and above	3333	37.17
Women’s level of education		
No education	3333	37.16
Primary	1482	16.53
Secondary and above	4154	46.31
Husband/partner’s level of education		
No education	3074	34.27
Primary	1199	13.36
Secondary and above	4697	52.37
Marital status		
Currently married	8128	90.63
Cohabitating	337	3.76
Previously married	504	5.62
Working status		
No	2497	27.84
Yes	6472	72.16
Ethnicity		
Hausa	3046	33.97
Yoruba	1587	17.70
Igbo	1391	15.51
Others	2944	32.83
Religion		
Christianity	4248	47.36
Islam	4667	52.03
Traditionalist and others	54	0.60
Parity		
0	604	6.73
1–3	4652	51.87
4 and above	3713	41.40
Exposure to media		
No	2786	31.06
Yes	6183	68.94
Place of residence		
Urban	4064	45.31
Rural	4905	54.69
Wealth index		
Poorest	1602	17.86
Poorer	1726	19.25
Middle	1827	20.37
Richer	1862	20.76
Richest	1952	21.76
Region		
North Central	1267	14.12
North East	1304	14.53
North West	2434	27.14
South East	1070	11.93
South South	1024	11.41
South West	1871	20.86
Sex of household head		
Male	7735	86.24
Female	1234	13.76
Community literacy level		
Low	3019	33.66
Medium	2826	31.51
High	3124	34.83
Community socioeconomic status		
Low	5131	57.21
Medium	623	6.95
High	3215	35.85

NDHS, 2018

## Spatial analysis results

### Spatial distribution of IPV in Nigeria

**Fig. 1:**
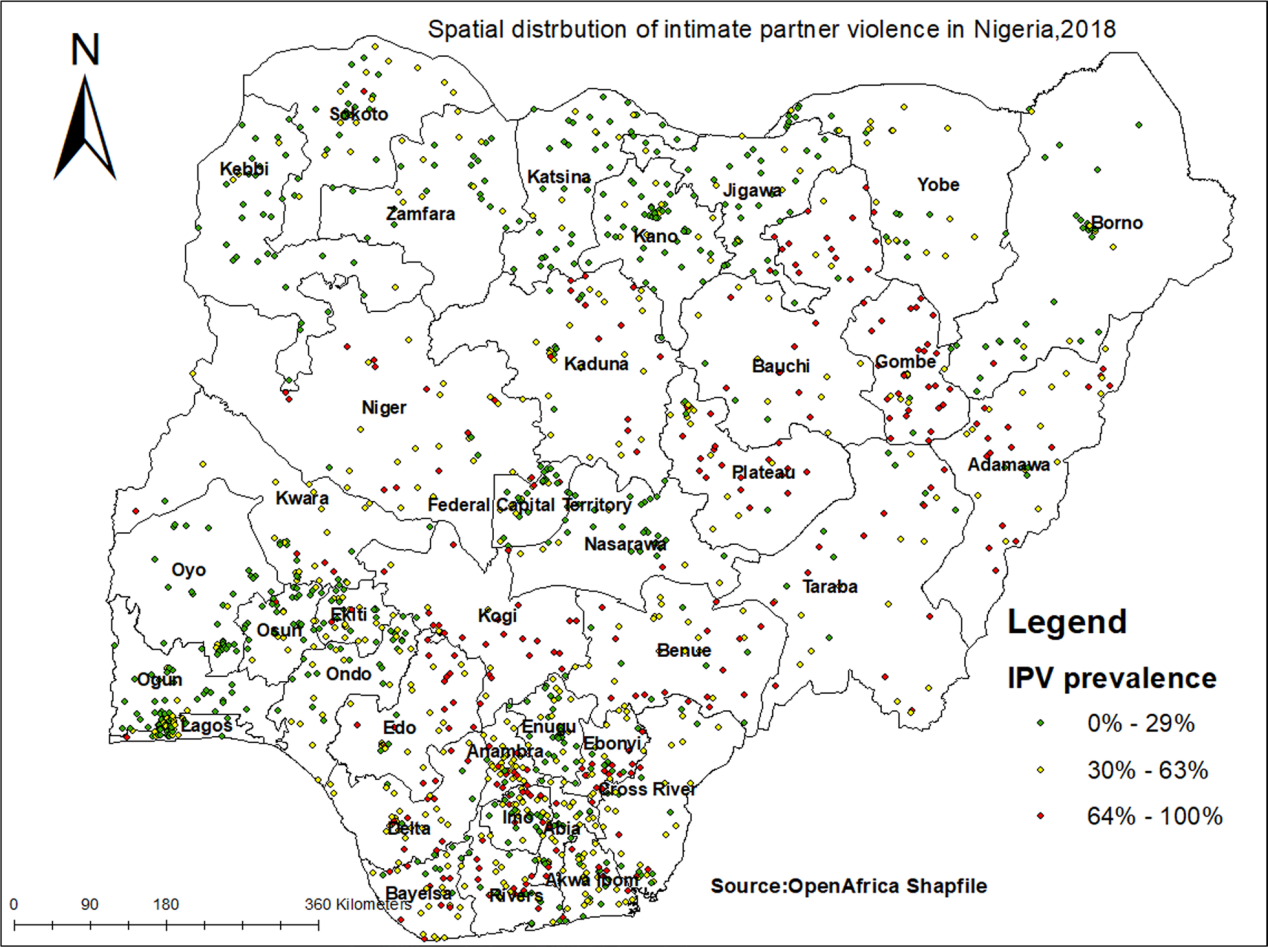
Geographical location of IPV among women in Nigeria, 2018

Out of 1400 total clusters, 1,393 clusters were used for spatial analysis of IPV in Nigeria. The Enumeration areas on the Nigeria map is located by points. Each enumeration area had a proportion of IPV ranges from zero to hundred percent. High proportion of IPV were represented by red colors whereas the low proportion of IPV were represented by green colors (Fig. 1).

### Spatial autocorrelation

**Fig. 2:**
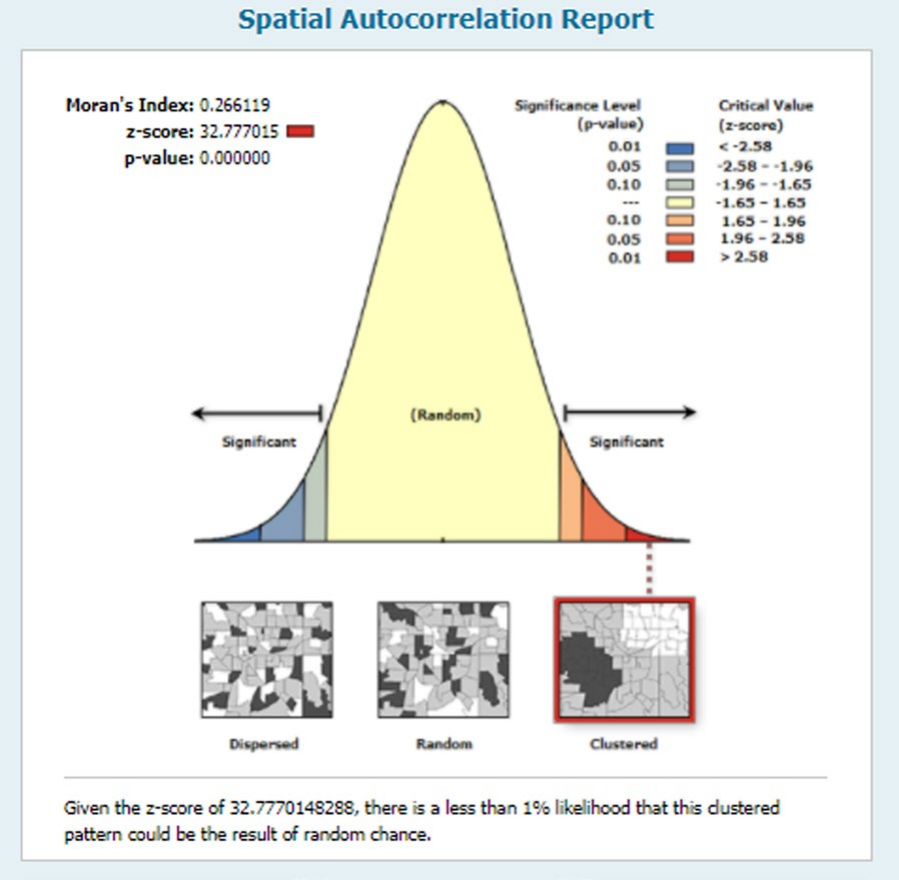
The autocorrelation spatial result of IPV reproductive age women in Nigeria, 2018

The spatial autocorrelation analysis was done using ArcGIS software to check whether there is clustering effect on IPV distribution in Nigeria. The statistical analysis results found were Global Moran’s I = 0.26, *p *≤ 0.001 and Z-score 32.77. This indicates that there is clustering effect in IPV distribution in Nigeria (Fig. 2).

### Hot spot analysis of IPV in Nigeria

**Fig. 3:**
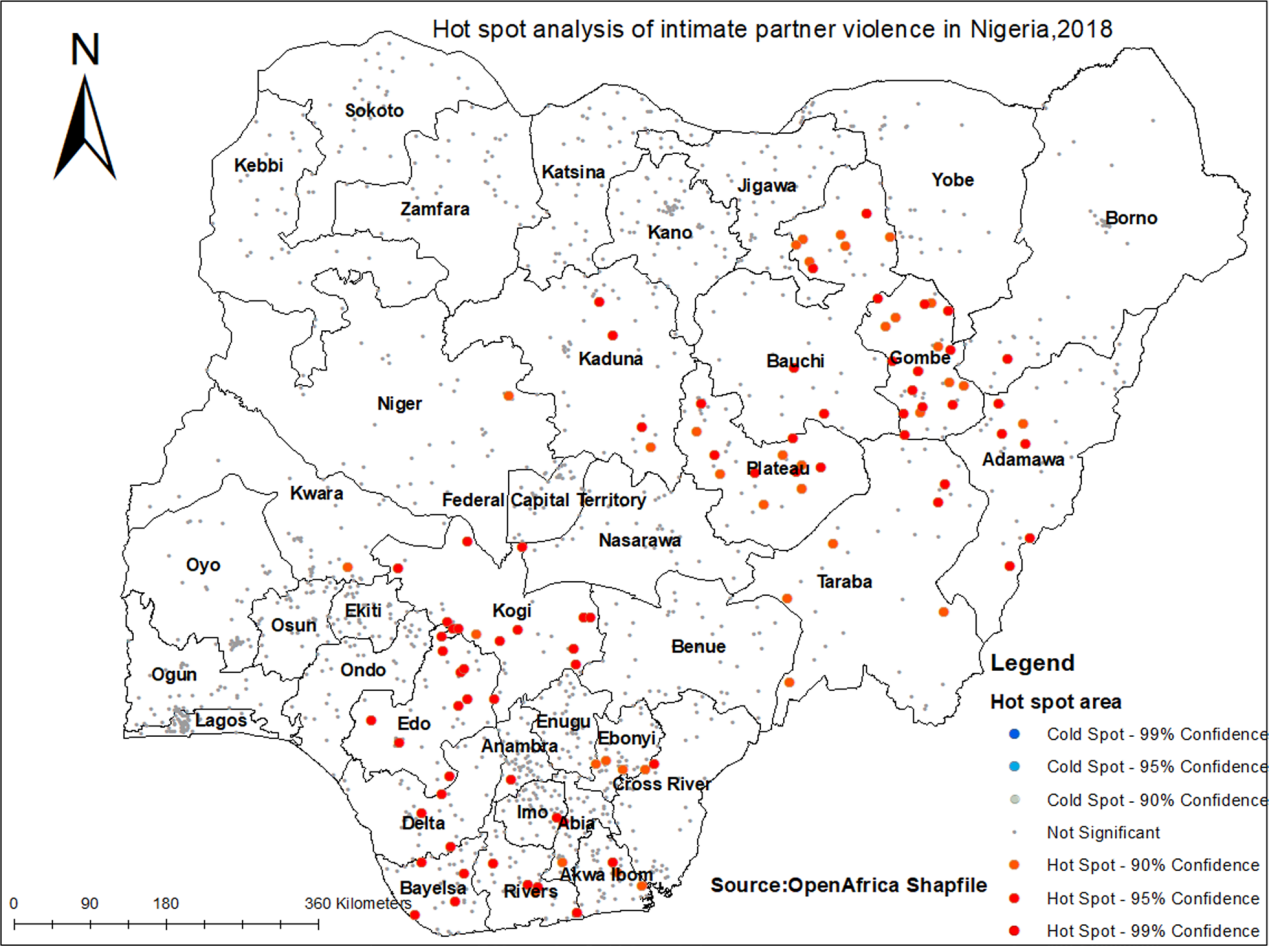
Hot spot analysis of IPV among women in Nigeria, 2018

The hot spot analysis revealed a high proportion of women who faced IPV (hot spot) and low-proportion women who faced IPV (cold spot area). Risk areas were represented by red colors (high rate of IPV). The hot spot areas were located in Gombe, Bauchi, Adamawa, Plateau, Kogi, Edo, Ebonyi, and Rivers (*p* < 0.010) (Fig. 3).

### Prediction of IPV in Nigeria

**Fig. 4:**
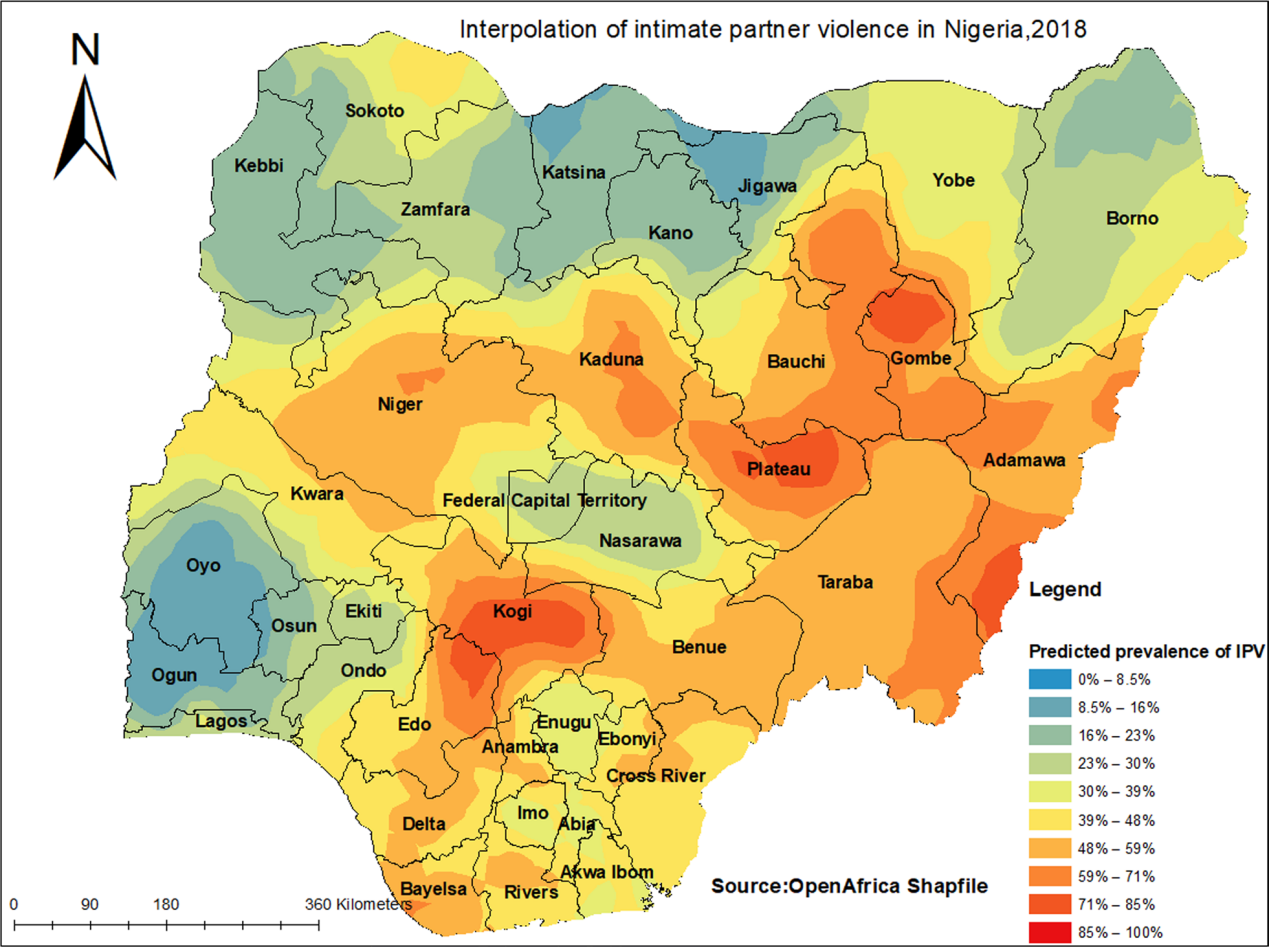
Prediction of IPV among reproductive age women in Nigeria, 2018

Travel from blue to red-colored areas, interpolated IPV over the area increases, red color reveals the predicted IPV risk area maps, and blue color reveals the predicted low IPV risk areas. The high predicted IPV areas were located in Kogi, Plateau, Kaduna, Adamawa, Gombe, and Niger. The low predicted IPV areas were located in Oyo, Ogun, Lagos, Katsina, and Jigawa (Fig. 4).

### SaTScan analysis of IPV

**Fig. 5:**
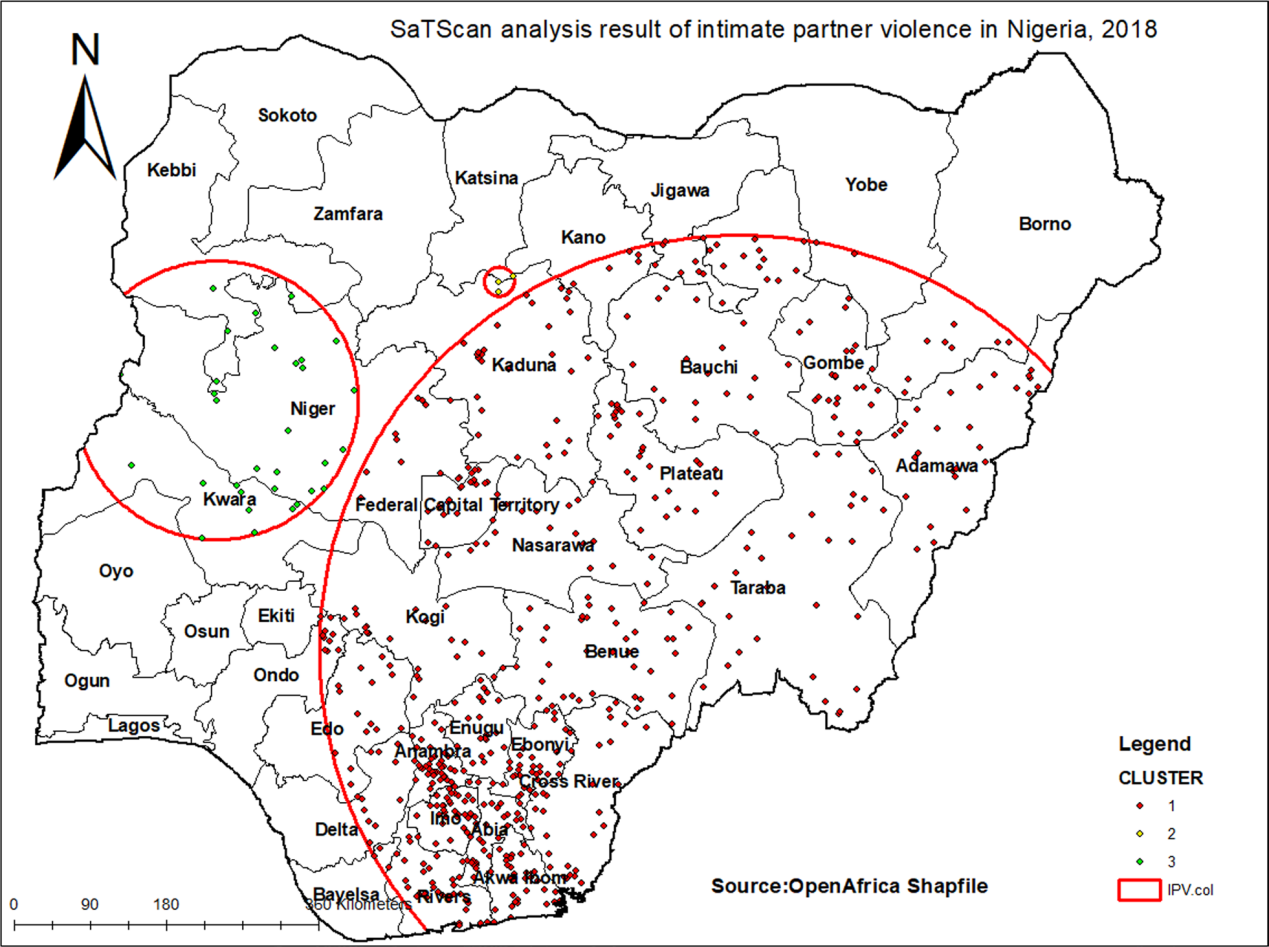
The SaTScan analysis result of IPV among women in Nigeria, 2018

Primary clusters of IPV were detected. 1095 primary significant clusters were located. Primary spatial windows were located in the Eastern and Southern parts of Nigeria, which located at (7.347914 N, 10.176090 E)/492.24 kilo meter radius, and LLR of 143 and RR 1.64 with significant *p *value. It was revealed that women within the spatial window had a 1.64 times higher risk of IPV than women outside the window. The secondary clusters spatial window was typically located in the Western part of Nigeria, but it was not significant (Table 2; Fig. 5).

**Table 2:** SaTScan analysis of IPV among women in Nigeria, 2018

Cluster	Enumeration area (cluster) identified	Coordinate/radius	Population	Case	RR	LLR	*P* value
1	1095	(7.347914 N, 10.176090 E)/492.24 km	3680	1825	1.64	143	< 0.001

### Multi-level fixed effects (measures of associations) results

The significant predictors at the individual level were women's education, marital status, working status, ethnicity, religion, parity, and mass media exposure. The likelihood of experiencing IPV among women in Nigeria was higher among women with primary education [aOR = 1.32; 95%(CI = 1.09–1.60)], those that were previously married [aOR = 1.73; 95%(CI = 1.33–2.25)], women currently working [aOR = 1.29; 95%(CI = 1.14–1.47)], women who were Yoruba [aOR = 1.39; 95%(CI = 0.99–1.94)], women with parity from four and above [aOR = 1.78; 95%(CI = 1.40–2.25)], women exposed to mass media [aOR = 1.41;95%(CI = 1.00–1.31)], compared to women who were without education, women that were not yet married, women not currently working, women whose ethnicity was Hausa, women without any children and those who were not exposed to mass media. Lower odds of IPV were reported among women who were practicing Islam [aOR = 0.47; 95%(CI = 0.47–0.69)] compared to those practicing Christianity (Table 3).

**Table 3:** Multilevel logistic regression models for individual and household/community predictors of intimate partner violence in Nigeria

Variables n = 8968	Model 0	Model I	Model II	Model III
		aOR[95% CI]	aOR[95% CI]	aOR[95% CI]
*Fixed effects results*				
Individual-level variables				
Age of respondent				
15–24		RC		RC
25–34		1.05[0.90–1.22]		1.12[0.97–1.31]
35 and above		0.79**[0.67–0.94]		0.89[0.74–1.06]
Women’s level of education				
No education		RC		RC
Primary		0.95[0.79–1.13]		0.99[0.84–1.19]
Secondary and above		0.89[0.74–1.06]		1.05[0.87–1.27]
Husband/Partner’s level of education				
No education		RC		RC
Primary		1.27*[1.05–1.53]		1.32**[1.09–1.60]
Secondary and above		0.93[0.79–1.11]		1.04[0.87–1.24]
Marital status				
Currently married		RC		RC
Cohabitating		1.13[0.87–1.48]		1.20[0.91–1.56]
Previously married		1.72***[1.35–2.19]		1.73***[1.33–2.25]
Working status				
No		RC		RC
Yes		1.30***[1.14–1.47]		1.29***[1.14–1.47]
Ethnicity				
Hausa		RC		RC
Yoruba		0.70**[0.54–0.90]		1.39*[0.99–1.94]
Igbo		1.09[0.83–1.43]		1.01[0.70–1.47]
Others		1.52***[1.25–1.84]		1.10[0.89–1.37]
Religion				
Christianity		RC		RC
Islam		0.65***[0.54–0.90]		0.57***[0.47–0.69]
Traditionalist and others		0.52*[0.28–0.96]		0.55*[0.30–1.01]
Parity				
0		RC		RC
1–3		1.49***[1.19–1.85]		1.47**[1.18–1.84]
4 and above		1.89***[1.49–2.39]		1.78***[1.40–2.25]
Exposure to mass media				
No		RC		RC
Yes		0.99[0.87–1.12]		1.14*[1.00–1.31]
*Household-level*				
Place of residence				
Urban			RC	RC
Rural			0.88[0.74–1.05]	0.85[0.72–1.01]
Wealth index				
Poorest			RC	RC
Poorer			0.82*[0.69–0.98]	0.78**[0.65–0.93]
Middle			0.76**[0.62–0.92]	0.71**[0.58–0.87]
Richer			0.65***[0.52–0.81]	0.61***[0.48–0.78]
Richest			0.47***[0.36–0.62]	0.45***[0.34–0.61]
Region				
North Central			RC	RC
North East			1.11[0.87–1.41]	1.50**[1.16–1.92]
North West			0.31***[0.25–0.39]	0.49***[0.37–0.64]
South East			0.94[0.73–1.21]	0.75[0.51–1.10]
South South			1.27*[0.99–1.63]	1.01[0.78–1.31]
South West			0.33***[0.25–0.42]	0.25***[0.18–0.34]
Sex of household head				
Male			RC	RC
Female			1.18*[1.02–1.36]	1.01[0.86–1.20]
Community literacy level				
Low			RC	RC
Medium			1.05[0.87–1.27]	0.89[0.73–1.09]
High			1.04[0.82–1.33]	0.83[0.65–1.07]
Community socioeconomic status				
Low			RC	RC
Medium			1.42*[1.03–1.96]	1.45*[1.06–2.00]
High			0.92[0.73–1.15]	0.97[0.77–1.21]
Random effects results				
PSU Variance (95% CI)	1.23[1.06–1.44]	1.00[0.84–1.18]	0.84[0.71–1.01]	0.81[0.68–0.98]
ICC	0.27	0.23	0.20	0.19
LR test	χ2 = 658.59, *p* < 0.001	χ2 = 472.39, *p* < 0.001	χ2 = 369.95, *p* < 0.001	χ2 = 342.95, *p* < 0.001
Wald χ2	Reference	237.68***	318.78***	450.12***
Model fitness				
Log-likelihood	− 5581.21	− 5461.92	− 5422.82	− 5348.14
AIC	11,166.42	10,961.84	10,879.64	10,764.27
Number of clusters	1383	1383	1383	1383

Weighted NDHS, 2018

Exponentiated coefficients; 95% confidence intervals in brackets

Model 0 is the null model, a baseline model without any determinant variable

Model I is adjusted for individual-level variables (Age of respondent, women educational level, spouse educational level, marital status, currently working, ethnicity, religion, parity, and media exposure)

Model II is adjusted for household/community level variables (Place of residence, wealth index, region, sex of household head, community literacy level, community socioeconomic status)

Model III is the final model adjusted for individual and household/community level variables

AOR, adjusted odds ratios; CI, confidence interval; RC, reference category; PSU, primary sampling unit; ICC, intra-class correlation; LR test, likelihood ratio test; AIC, Akaike’s information criterion

*p < 0.05; **p < 0.01; ***p < 0.001

At the household/community level, the significant predictors were wealth index, region, and community socioeconomic status. Women who resided in North East [aOR = 1.50;95%(CI = 1.16–1.92)], and in communities with medium socioeconomic status [aOR = 1.45;95%(CI = 1.06–2.00)], were more likely to experience IPV compared to those residing in the North Central and in communities with low socioeconomic status. Women who were within the richest wealth index [aOR = 0.45; 95%(CI = 0.34–0.61)], and those residing in the South West region [aOR = 0.25; 95%(CI = 0.18–0.34)] were less likely to experience IPV compared to those of the poorest wealth index and those residing in North Central (Table 3).

### Random effects (measures of variations) results

The empty model (Model 0), as shown below in Table 3, depicted a substantial variation in the likelihood of IPV among women in Nigeria across the clustering of the Primary Sampling Units (PSUs) [σ2 = 1.23; 95%(CI = 1.06–1.44)]. The “model 0” indicated that 27% of the variation in IPV among women in Nigeria was attributed to the variation between Intra-Class Correlation, i.e., (ICC = 0.27). The between-cluster variation decreased to 23% (0.23) in Model I (individual level only). In the household/community-level only (Model II), the ICC decreased further to 20%, while the ICC declined to 19% in the complete model with both the individual and household/community factors (Model III). This further reiterates that the variations in the likelihood of IPV in Nigeria are attributed to the variation in PSUs. The Akaike’s Information Criterion (AIC) values showed a successive reduction, which means a substantial improvement in each of the models over the previous model and also affirmed the goodness of fit of Model III. Therefore, Model III, the complete model with both the selected individual and household/community factors, was selected to predict the likelihood of IPV among women in Nigeria (Table 1).

## Discussion

The study examined the spatial distribution and predictors of IPV among women in Nigeria using the recent NDHS data conducted in 2018. We found that the lower and higher proportions of IPV ranged from 0 to 29% and 64% to 100%, respectively. The high predicted IPV areas were located in Kogi, Plateau, Kaduna, Adamawa, Gombe, and Niger, whereas the low predicted IPV areas were located in Oyo, Ogun, Lagos, Katsina, and Jigawa. A possible reason for this finding could be the insurgency of Boko Haram, which increased the rates of violence against women in the conflict-affected areas (i.e., Northeastern states) [33]. It is also possible that the Boko Haram insurgency might have reduced the household autonomy of women by not engaging in any socioeconomy activities, thereby increasing their susceptibility to IPV [34]. The finding indicates that conflicts play a significant role in the rate of IPV against women. Therefore, efforts to reduce the rate of IPV among women in Nigeria should consider preventing the occurrences of conflicts and empowering women as this will help curtail the surge in IPV rates among women residing in the Northeastern region of the country.

The study also found that women who were previously married were more likely to experience IPV. The likely explanation for this finding could be that women who were previously married were abused, leading to their decision to opt-out of the marriage [35]. Additionally, perhaps these women believed that once they have been abused, there is a likelihood that their spouse would continue to abuse them; hence, leaving the marriage becomes the best option for them.

Corroborating the findings of other previous studies [36, 37], the study found that Yoruba were more likely to experience women IPV than women from the Hausa ethnic group. A possible reason for this finding could be that women from the Hausa ethnic group see violence against them as a norm, resulting in their reduction in the report rates of IPV perpetrated against them [36].

Women with parity from four and above and those exposed to mass media were more likely to experience IPV. The reasons for these findings could be that women who have four and above children with a male spouse may depend solely on the spouse for their needs, increasing their susceptibility to experiencing IPV [38]. It could also be that women exposed to mass media have been educated; hence, they are more empowered to fight for their rights, exposing them to be abused [39].

Similar to other previous studies by Ahinkorah et al., [17] and Memiah et al. [34], lower odds of IPV were reported among women who were practicing Islam compared to those practicing Christianity. A possible reason for this finding could be the existence of certain strict norms in the Islamic religion regarding how diligently women should be treated, decreasing the likelihood of Islamic women experiencing IPV [17]

Women who reside in North East and medium community socioeconomic status were more likely to experience IPV compared to those residing in the North Central and community with low socioeconomic status. Probably, the low level of socioeconomic status of women residing in the North East region of Nigeria predisposes them to be abused by their spouses [40]. Therefore, it is valid to assume that increasing the socioeconomic status of women protects them from being abused.

Akin to other previous studies [17, 41], women within the richest wealth index and those residing in the South West region were less likely to experience IPV compared to those residing in the South West region who were poorest and those residing in North Central. A possible reason for this finding could be that women who are from rich households are more empowered and have access to needed resources, and they can help fight for their rights and the rights of other marginalized women in the communities, reducing their chances of been abused [17, 41]. It is also possible that women residing in the South West region of Nigeria are more empowered than their counterparts in the North Central region of Nigeria, protecting them from been abused [42].

### Implications for public health policy and future research

The findings of this study are relevant to policy and public health. The inequality in IPV experience based on wealth index, favouring women who belonged to the richest wealth quintile, calls for the attention of policymakers and key stakeholders in policy formulation and implementation to have policies on IPV that are pro-poor. Emphasis must be placed on the poor's special needs to empower them and eliminate the macro-level factors like poverty that permeate IPV perpetration. Public health-wise, the findings identified the hotspots of IPV and mapped out the spatial distribution of IPV. This should provide public health providers with the blueprint that will guide their programs and interventions. Furthermore, it will help to ensure optimal utilization of scarce resources in the fight against IPV in Nigeria, as priority will be given to provinces that are hotspots for IPV. Future research could seek to explore how in-depth Nigeria women wealth quintile can further be used in fighting against IPV in Nigeria by conducting qualitative research among the local communities.

### Strengths and limitations

Our study has several strengths. First, the use of nationally representative data boosts the capacity of our findings to be generalized to women in Nigeria. Additionally, the use of Geographical Information System (GIS) in the analysis of the spatial distribution enabled us to identify the hotspots of IPV in Nigeria, and this is a major contribution to the web of literature on IPV in Nigeria. Moreover, identifying these IPV hotspots would be beneficial to program designers and implementers in their design on context-specific and population-targeted interventions to alleviate IPV. Nevertheless, the study was not without some limitations. A major limitation to this study was that the data used was cross-sectional in design, limiting us from establishing causality. Also, the data was self-reported, making it highly susceptible to recall bias and social desirability bias since IPV in itself is not socially acceptable.

## Conclusion

The study found regional variations in the prevalence of IPV among women in Nigeria. The high prevalent IPV areas were located in Kogi, Plateau, Kaduna, Adamawa, Gombe, and Niger, whereas the low prevalent IPV areas were located in Oyo, Ogun, Lagos, Katsina, and Jigawa. Further, the study has identified the individual and community level factors that predict IPV perpetration among Nigerian women. Therefore, policymakers should consider the factors identified in this study to reduce IPV prevalence among women in Nigeria. Chiefly, empowering women would yield a significant improvement in the fight against gender-based violence.

## Data Availability

The datasets utilized in this study can be accessed at https://dhsprogram.com/data/available-datasets.cfm.

## Abbreviations

IPV: Intimate partner violence
NDHS: Nigeria Demographic Health Survey
WHO: World Health Organization
UN: United Nations
SDGs: Sustainable Development Goals
FCT: Federal Capital Territory
NPC: National Population Commission
SSA: Sub-Saharan
LLR: The log-likelihood ratio
AIC: Akaike information criteria
VIF: Variance inflation factor
EAs: Enumerations areas
RR: Relative risk
ICC: Intra-class correlation
GIS: Geographical information system
